# Chirality-Induced Spin Selectivity at the Molecular Level: A Different Perspective to Understand and Exploit the Phenomenon

**DOI:** 10.1021/acs.jpclett.5c00755

**Published:** 2025-05-21

**Authors:** Alessandro Chiesa, Alberto Privitera, Elena Garlatti, Giuseppe Allodi, Robert Bittl, Michael R. Wasielewski, Roberta Sessoli, Stefano Carretta

**Affiliations:** † Università di Parma, Dipartimento di Scienze Matematiche, Fisiche e Informatiche, I-43124 Parma, Italy; ‡ INFN-Sezione di Milano Bicocca, Gruppo Collegato di Parma, 43124 Parma, Italy; § Consorzio Interuniversitario Nazionale per la Scienza e Tecnologia dei Materiali (INSTM), I-50121 Firenze, Italy; ∥ Department of Chemistry, Center for Molecular Quantum Transduction, and Institute for Quantum Information Research and Engineering, 3270 Northwestern University, Evanston, Illinois 60208-3113, United States; ⊥ Dipartimento di Ingegneria Industriale, Università degli Studi di Firenze, I-50139, Firenze, Italy; # Freie Universität Berlin, Fachbereich Physik, Berlin Joint EPR Lab, D-14195 Berlin, Germany; ∇ Dipartimento di Chimica “U. Schiff” (DICUS), Università degli Studi di Firenze, I-50019 Sesto Fiorentino, Firenze, Italy

## Abstract

Investigating Chirality-Induced Spin Selectivity (CISS) at the molecular level offers a novel perspective, in between Chemistry and Physics, on this still not fully understood phenomenon. Indeed, the molecular approach offers an advantage point for understanding CISS by disentangling the role of chiral molecules from that of the surfaces. Here, we present an overview of experimental observations of CISS in electron transfer on isolated molecules in solution and the current status of theory to model the phenomenon. We discuss what is accomplished and which are the most important questions, and we propose experiments based on electron and nuclear magnetic resonance both to unravel open issues on the CISS effect in electron transfer and to apply it to quantum technologies.

## Graphical abstract

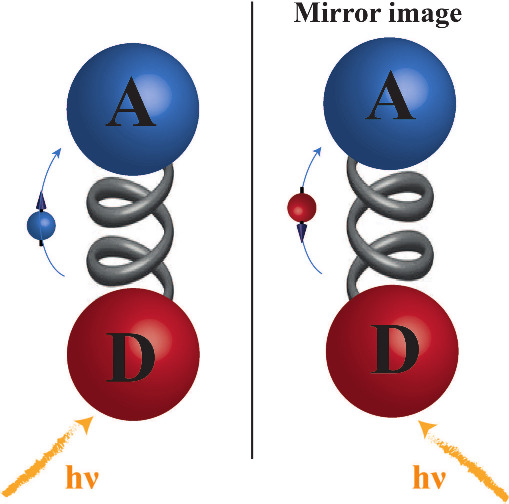

Research on Chirality-Induced Spin Selectivity (CISS) effect is growing rapidly^1^ and attracts the attention of a multidisciplinary community, spanning from Chemistry to Physics and Materials Science. The CISS effect refers to a rather wide class of experiments in which electrons passing through a chiral medium are spin polarized or spin filtered. By linking the spin direction to the molecular orientation, CISS could have important implications for biological processes and chemical reactions,^2–4^ besides opening new avenues for interesting applications in spintronics^5^ and quantum technologies.^6,7^

Evidence of CISS has been reported in very different experiments, most of which can be grouped into four broad categories:^1,8^ (i) photoelectron spectroscopy,^9–11^ (ii) electron transport,^12–14^ (iii) charge/spin polarization after deposition on a surface,^15–17^ and (iv) spectroscopic methods.^18–22^ In (i), a layer of chiral molecules is placed on a metal substrate. Then, electrons from the substrate are photoexcited and scattered at different angles depending on their spin orientation after having crossed the chiral material. This method is considered the technique of choice to probe CISS, since the normalized difference in the number of electrons scattered at the two detectors directly gives the spin polarization, without involving current.^1,11^ Spin polarization in the order of 30–40% was reported for DNA, oligopeptides, oligonucleotides, and helicene.^11^

In typical transport experiments (ii), a chiral monolayer is placed between two electrodes (or between an electrode and an atomic force microscopy tip) of which one is a ferromagnet (FM) and the current is measured for the two opposite spin polarizations of the injected/detected current. The difference observed in the current–voltage *I*(*V*) curves for the two cases indicates a spin-filtering/polarization effect of the chiral layer and is assumed to measure the corresponding spin selectivity. The latter can reach remarkably high values, on the order of 50–60% for DNA (increasing with the molecular length^23^), even 90% in supramolecular wires or 2D chiral hybrid lead-iodide perovskites^24^ and copper halides^25^ and almost 100% in metal–organic frameworks^26^ or Al_2_O_3_/organic hybrid films.^27^ CISS was also observed in transport through a single-molecule break junctions with scanning tunneling or atomic-force microscopy,^14,28^ reaching polarization as high as 80% for helicene.^28^

The third class of experiments (iii) involves chiral molecules in a static, out-of-equilibrium, configuration. These measurements include magnetization switching by enantiomer deposition,^16^ selection of enantiomer adsorption by the polarization of a FM substrate,^17^ or Kelvin probe measurements on FM electrodes covered by a chiral monolayer. These experiments indicate that a strong interaction exists between chiral molecules and a FM substrate and suggest that penetration of the electron spin wave function in the chiral layer is enantiospecific.^30^ In addition, a Hall potential was detected in a two-dimensional electron gas coated with a chiral monolayer subject to an electric field,^15,31^ suggesting a spin polarization accompanying the charge polarization.^32^

In most of these studies, molecules were placed on metal surfaces, thus pointing to a possible decisive role of the large spin–orbit coupling of the substrate in establishing a spin polarization.^33–35^ Recently, CISS was also observed in isolated molecules dispersed in solution using spectroscopic methods (iv). Among these, time-resolved electron paramagnetic resonance (TREPR) gives direct access to the electron spin degrees of freedom, providing a valuable approach for investigating CISS at the molecular level.^18,19^

Notably, the consistent feature across most of experimental observations of CISS, despite involving both bound and unbound particles in diverse environments,^36^ is the presence of organic chiral molecules composed of carbon and other light atoms with weak spin–orbit coupling. This poses a significant challenge for theoreticians, who are working to develop a comprehensive explanation.^36^

Important steps toward a deeper understanding of CISS can be gained by disentangling different players in the aforementioned approaches and focusing only on isolated chiral molecules undergoing photoinduced electron transfer (PET). This is also the situation which more closely resembles biological processes and that opens perspectives for exploiting CISS not only in spintronics^5^ but also in quantum technologies, where CISS might enable spin control and readout at the single-molecule level.^6–8,37^ Therefore, motivated by these exciting applications and by a fundamental interest in understanding this intriguing phenomenon, we focus here on the novel perspectives opened by studying the CISS in PET.

In this perspective, we first provide a brief introduction on PET and on the technique of choice to probe the spin dynamics in charge transfer systems, i.e., TREPR. Then, we illustrate how this technique can be exploited to reveal the CISS effect and discuss experimental observations in isolated molecules achieved so far. We then review current theoretical explanations, followed by a discussion of possible steps forward to improve our knowledge of CISS by combining more accurate theoretical models with targeted experiments to address open issues. These are based on exploiting either electronic or nuclear spins as a probe of the local spin polarization through EPR methods and broadband nuclear magnetic resonance (NMR). Finally, we discuss how the CISS effect in isolated molecules could be exploited as an important tool for quantum technologies, such as quantum computing and quantum sensing.

## Electron Transfer and Spin-Correlated Radical Pairs

Investigating the spin dynamics between electron donor (D) and acceptor (A) molecules linked by a chiral bridge after PET provides an ideal tool for studying the CISS effect at the molecular level. A scheme of the electron-transfer process is shown on the left side of Figure 1. We start with both D and A in their ground state, with doubly occupied HOMO orbitals. After light irradiation, an electron is photoexcited to a higher orbital, keeping the donor electron pair in a spin singlet. A spin-correlated radical pair (SCRP) is generated after ET, which is still a singlet for fast electron transfer through an achiral bridge. A triplet character of the SCRP can originate either from radical pair intersystem crossing, or from the CISS effect in the case of the chiral bridge, as discussed in the following section. A technique of choice to probe the out-of-equilibrium population and the spin dynamics of these SCRPs is TREPR.

**Figure 1:**
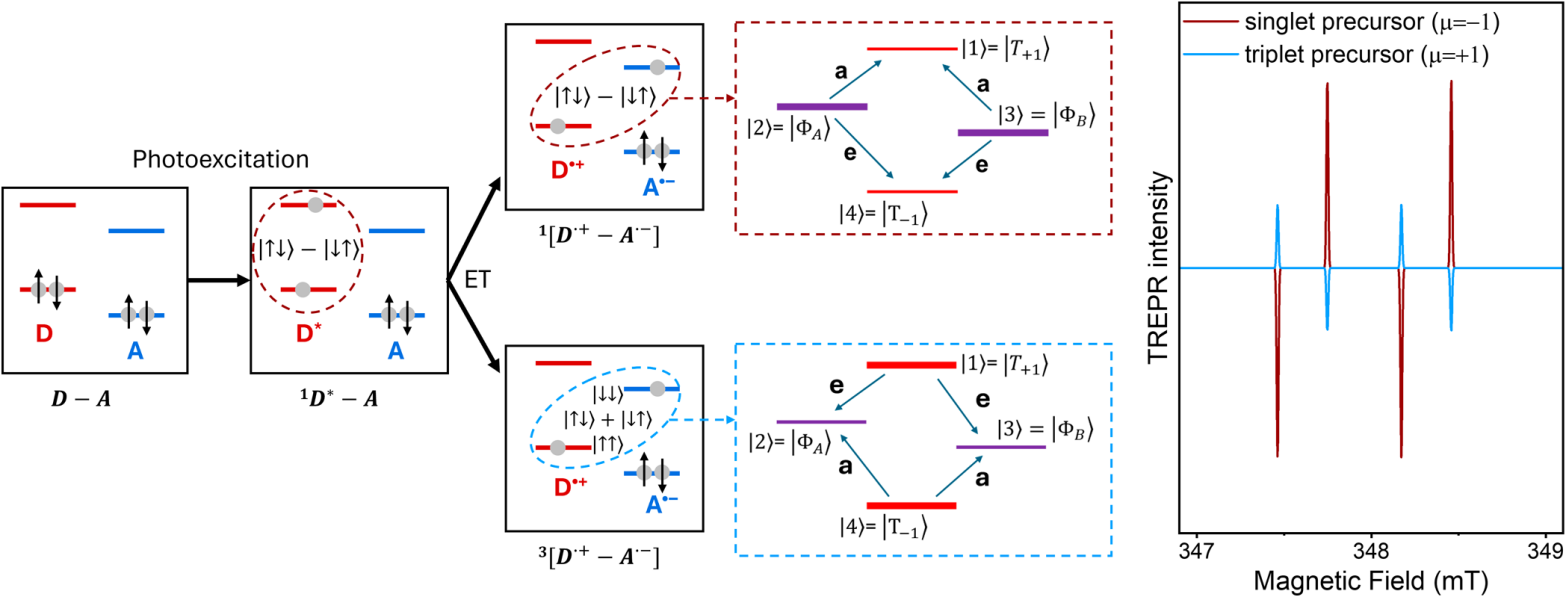
(left) Schematic representation of the electron transfer mechanism. The initial state is a singlet on the donor (D), with both electrons in the ground-state orbital. Photoexcitation promotes the system to the D*–A state. Following electron transfer (ET) of the excited electron to the acceptor, a spin-correlated radical pair (SCRP) is formed, which can be a singlet (top) or a triplet (bottom), as discussed in the text. (center, dotted square): SCRP energy levels in the high field limit for an ET starting from either a singlet or a triplet precursor with equal sublevel populations. Enhanced absorptive (a) and emissive (e) microwave-induced transitions are indicated. (right) Simulated TREPR spectra with the following spin Hamiltonian parameters: Δ*g* = 0.004, *J* = 0 MHz, and *D* = 4 MHz, θ = 0.

For magnetic fields making the Zeeman energy significantly larger than the spin–spin coupling between the two radicals of the pair, the eigenstates are the pure triplet states |*T*_+1_⟩ and |*T*_–1_⟩ and linear superpositions of |*S*⟩ and |*T*_0_⟩ labeled in the following as |Φ_*A*_⟩ = cos ϕ|*S*⟩ + sin ϕ|*T*_0_⟩ and |Φ_*B*_⟩ = cos ϕ|*T*_0_⟩ – sin ϕ|*S*⟩. Here, the angle ϕ defines the composition of the eigenstates and depends on the ratio between the difference in the Zeeman and hyperfine fields experienced by the two unpaired electrons and their respective exchange and/or dipolar couplings.^38^ We consider below EPR intensities computed in the linear-response regime and after loss of coherence between system eigenstates. Peak positions depend on differences between the eigenvalues of the spin Hamiltonian, while the intensities are determined by

1 $$I_{\mu \nu }\propto |\langle \mu |S_{y}{|\nu \rangle |}^{2}\left(p_{\mu }-p_{\nu }\right)$$

where |μ, ν⟩ = |*T*_±1_⟩, |Φ_*A*,*B*_⟩ are the Hamiltonian eigenstates, ⟨μ|*S*_*y*_|ν⟩ is the magnetic dipole matrix element of the |μ⟩ → |ν⟩ transition, and *p*_μ_ = |⟨μ|ψ_0_⟩|^2^ is the projection of the initial state |ψ_0_⟩ (i.e., the one just after the end of the ET process) onto eigenstate |μ⟩. Note that while peak positions depend only on the Hamiltonian eigenvalues, their intensities are modulated by the specific initial state. In particular, the sign of *I*_μν_ is determined by (*p*_μ_ – *p*_ν_), and it is positive if the initial state is more populated than the final one (i.e., *p*_μ_ > *p*_ν_), i.e., for absorption peaks (a), while it is negative in case of emission (e), which corresponds to *p*_μ_ < *p*_ν_.

As a result, the pattern of absorption–emission (ae) peaks in the TREPR spectrum depends (i) on the spin Hamiltonian parameters of the radical pair and (ii) on the precursor state, i.e., the spin of the photoexcited state that precedes ET (either singlet or triplet, as sketched in Figure 1). The spectra become particularly simple in the case of a pure singlet or a pure triplet precursor with equal sublevel populations, as shown in Figure 1 (right side). Then, the four allowed EPR transitions all have equal absolute intensity and give rise to either eaea or aeae patterns, depending on the sign of

2 $$\eta =\mu \mathrm{sign}\left[J-D\left(1-3\cos^{2}\theta \right)\right]$$

where μ = −1 (1) for singlet (triplet) precursor, *J* and *D* are the isotropic and dipolar spin–spin interactions between the two spins of the radical pair, and θ is the angle between the applied field and the dipolar axis (assumed coincident with the charge separation axis). Negative (positive) η is associated with eaea (aeae) pattern.^38–40^

If the two spins are separated by a distance *r* ≳ 2 nm, *J* can be neglected and *D* > 0 [a good estimate can be obtained in the point-dipole approximation]. Therefore, in an oriented sample with known θ, η is unequivocally determined by the precursor state, independently of the precise values of the other parameters, such as the *g* tensors of the two spins.

### Evidence of CISS by TREPR

In this context, the occurrence of the CISS effect can be detected through distinct TREPR fingerprints because the initial populations of the SCRP are strongly influenced by CISS. Consequently, if the PET precursor state is known, investigating the polarization pattern provides direct access to changes in the SCRP state due to CISS. To illustrate this concept, we consider a simple situation with a radical pair characterized by Δgμ_*B*_ *B* ≫ *D*, making eigenstates |Φ_*A*_⟩ and |Φ_*B*_⟩ practically factorized. Here, Δ*g* is the difference between the *g*–tensor components of the two spins along the applied field. Moreover, we consider both a chiral bridge (B_χ_) and an achiral reference (B) linked to the same donor and acceptor and hence described by the same spin Hamiltonian. This is the ideal configuration to highlight the CISS effect by comparing the TREPR response of DBA and DB_χ_A molecules. Indeed, for similar length of the two bridges, the only difference between chiral and achiral systems is the initial state, while the spin Hamiltonian parameters are fixed.

In particular, fast PET (<ns) does not allow intersystem crossing and hence results in a singlet-born SCRP and hence in a singlet charge-separated state for the achiral reference. Conversely, in simple models,^41,42^ the initial spin state of a CISS-polarized SCRP starting from a singlet precursor can be described as

3 $$|\psi \rangle =a|S\rangle +b|T_{0}\rangle$$

Here, the CISS effect produces a |*T*_0_⟩ component. For further considerations, it is useful to introduce the local spin polarization on the donor (*p*_D_ = 2⟨*s*_*z*D_⟩) and acceptor (*p*_A_ = 2⟨*s*_*z*A_⟩) both in the coupled (*S* – *T*_0_) and factorized basis. In particular, for a generic superposition state in the factorized basis |ψ⟩ = α|*↑↓*⟩ + β|*↓↑*⟩, we get

4 $$p_{D}\equiv 2\left\langle \psi \left|s_{zD}\right|\psi \right\rangle ={\left|\alpha \right|}^{2}-{\left|\beta \right|}^{2}\equiv -p_{A}$$

while in the coupled basis (in which |ψ⟩ = *a*|*S*⟩ + *b*|*T*_0_⟩), we obtain

5 $$p_{D}\equiv -p_{A}=Re\left[ab^{*}\right]$$

Hence, if ab* has a real component, CISS is accompanied by a local spin polarization, which is maximum when ab* is real and |*a*|^2^ = |*b*|^2^ = 1/2. Then, eq 3 reduces to |ψ_+_⟩ = |*↑↓*⟩ and |ψ_–_⟩ = |*↓↑*⟩. A purely real coherence ab* was first introduced by Luo and Hore,^43^ with $a=\cos\frac{\chi }{2}$ and $b=\sin\frac{\chi }{2}$ and – π/2 ≤ χ ≤ π/2. In this case, the CISS efficiency corresponds to $2\sin^{2}\frac{\chi }{2}$ . Due to the correspondence between the EPR spectra (sensitive to |*b*|^2^, see below), the same definition of CISS efficiency^19^ was also used referring to the model proposed by Fay^42^ and discussed in the theory section below, where the coherence ab* was purely imaginary (with no local spin polarization).

Figure 2 illustrates how the presence or absence of CISS manifests in the TREPR spectra. For a singlet-born SCRP in the absence of CISS (first column), the spectrum at θ = 0° consists of four transitions (eaea pattern) and is identical to that at θ = 180°. The effect of 100% CISS on the + enantiomer (middle column) results in the disappearance of two of the four peaks, the inner ones (for θ = 0°) or the outer ones (for θ = 180°). Indeed, if the eigenstates |Φ_*A*,*B*_⟩ are almost factorized, the CISS effect practically populates only one of them and the polarization is reversed by reversing the molecule (and thus the direction of the ET) with respect to the external field. Changing from θ = 0° to 180° is equivalent to changing the enantiomer. As a result, the sum of the spectra at θ = 0° and θ = 180° is identical for the two enantiomers. Nevertheless, they differ in intensity from the pure singlet case since the population difference of the eigenstates changes in the two cases, dependent on the mixing angle ϕ. The intensities for the two scenarios (singlet vs pure CISS) converge with decreasing spin–spin coupling or increasing Δ*g*μ_*B*_ *B* as in the example of Figure 2, where Δ*g*μ_*B*_ *B* ≫ *D*. Conversely, at θ = 90° or θ = 270° (red lines), the spectra for both enantiomers are identical but very different for chiral and achiral systems, with distinct spectral features arising from CISS. Indeed, in this case, two of the four peaks are reversed (eeaa pattern instead aeae in the absence of CISS).

**Figure 2:**
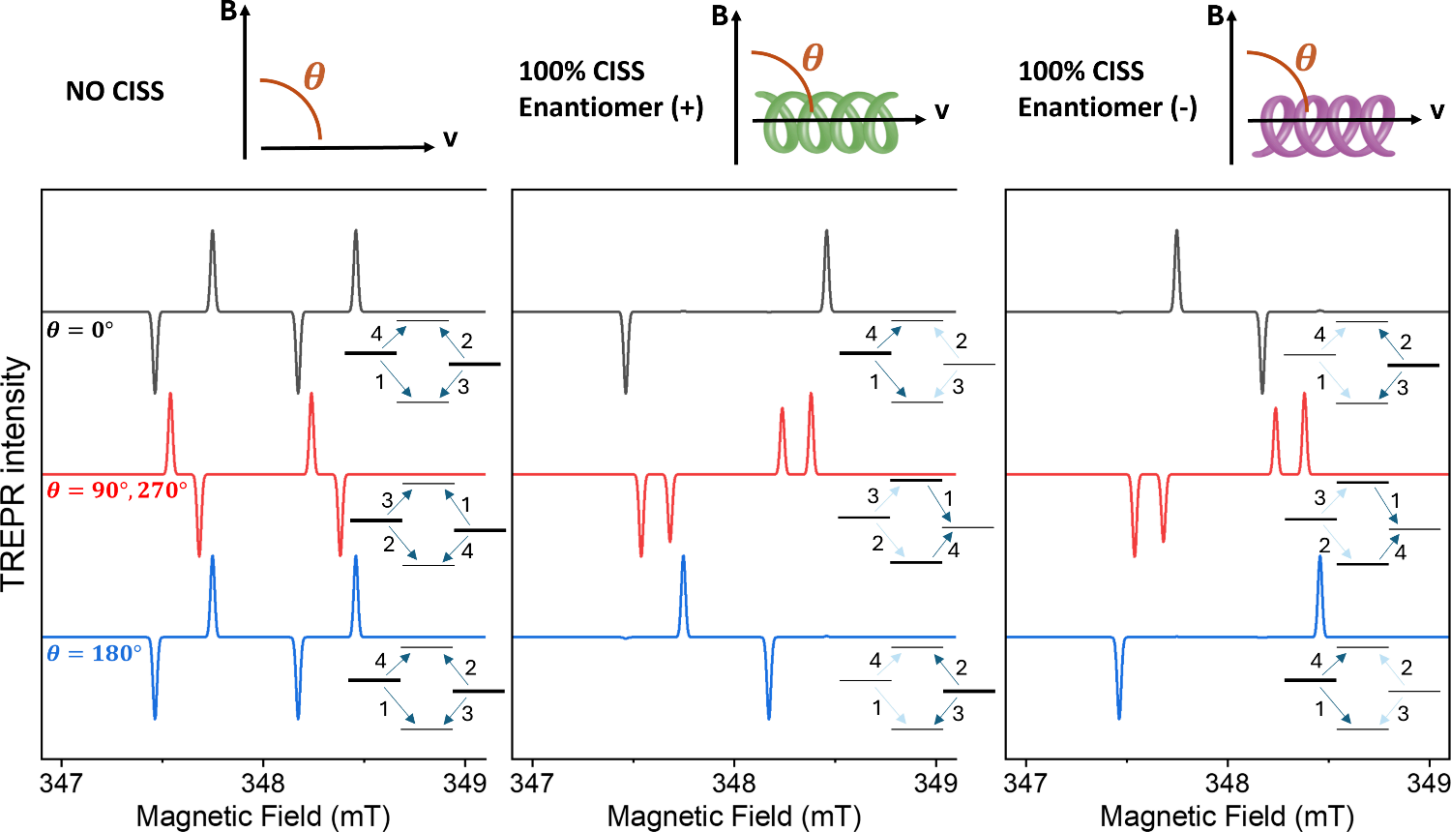
Simulated, normalized TREPR spectra of an SCRP with singlet precursor at selected orientations of the electron-transfer direction (θ), using the following spin Hamiltonian parameters: Δ*g* = 0.004, *J* = 0 MHz, and *D* = 4 MHz. For each simulation, the SCRP energy levels in the high-field limit and their relative populations are also reported. The numbers indicate EPR transitions in the spectrum from low to high field.

So far, we have discussed the ideal situation of 100% CISS efficiency and of an absolutely oriented sample. In most experiments, lower CISS efficiencies are observed, resulting in TREPR spectra with intermediate polarization patterns. Additionally, thus far, measurements to evidence CISS have not been performed in absolutely oriented samples but in liquid crystals. This inherently includes contributions from both orientations (θ = 0° and θ = 180°), resulting in a combined signal that cannot discriminate the two enantiomers and cannot be distinguished from that of a singlet for the longitudinal orientation of the external field, since the spectra for the (+)-enantiomer at θ = 0° coincides with that of the (−)-enantiomer at θ = 180° and vice versa. Nonetheless, any direction of the external field θ ≠ 0, 180° (and in particular θ = 90°) provides indications on the occurrence of CISS and hence also spectra collected on randomly oriented solutions can be informative^44^ in the presence of an anisotropic (e.g., dipolar) spin–spin interaction (see below), but without distinction of the two enantiomers. Future experimental directions to address this point are discussed below.

## Experimental Observation of CISS

From Figure 2, it emerges that a good way to evidence the occurrence of CISS is to study the orientation dependence of the EPR spectrum. Indeed, the chiral bridge introduces an axial anisotropy, which makes the initial state of the SCRP very different from that of the singlet, and the resulting spectrum is strongly dependent on the magnetic field orientation.

The first experimental observation of CISS in PET was achieved by orienting DB_χ_A molecules in a nematic liquid crystal (5CB).^18^ A sketch of the expected spectra for different orientations of the molecule with respect to the external field is shown in Figure 3a. Note that the four eaea or aeae peaks of Figure 2 are reduced to broadened ea or ae patterns because of similar *g*–tensors. As discussed above, the singlet case is isotropic and shows the same relative initial populations on |Φ_*A*_⟩ and |Φ_*B*_⟩ independently of θ. For longitudinal orientation of the field relative to the chiral axis (θ = 0°, 180°), perfect CISS leads to a similar excess of populations and hence singlet and 100% CISS states give the same pattern and are practically indistinguishable [see sketch in left panel of Figure 3a].

**Figure 3:**
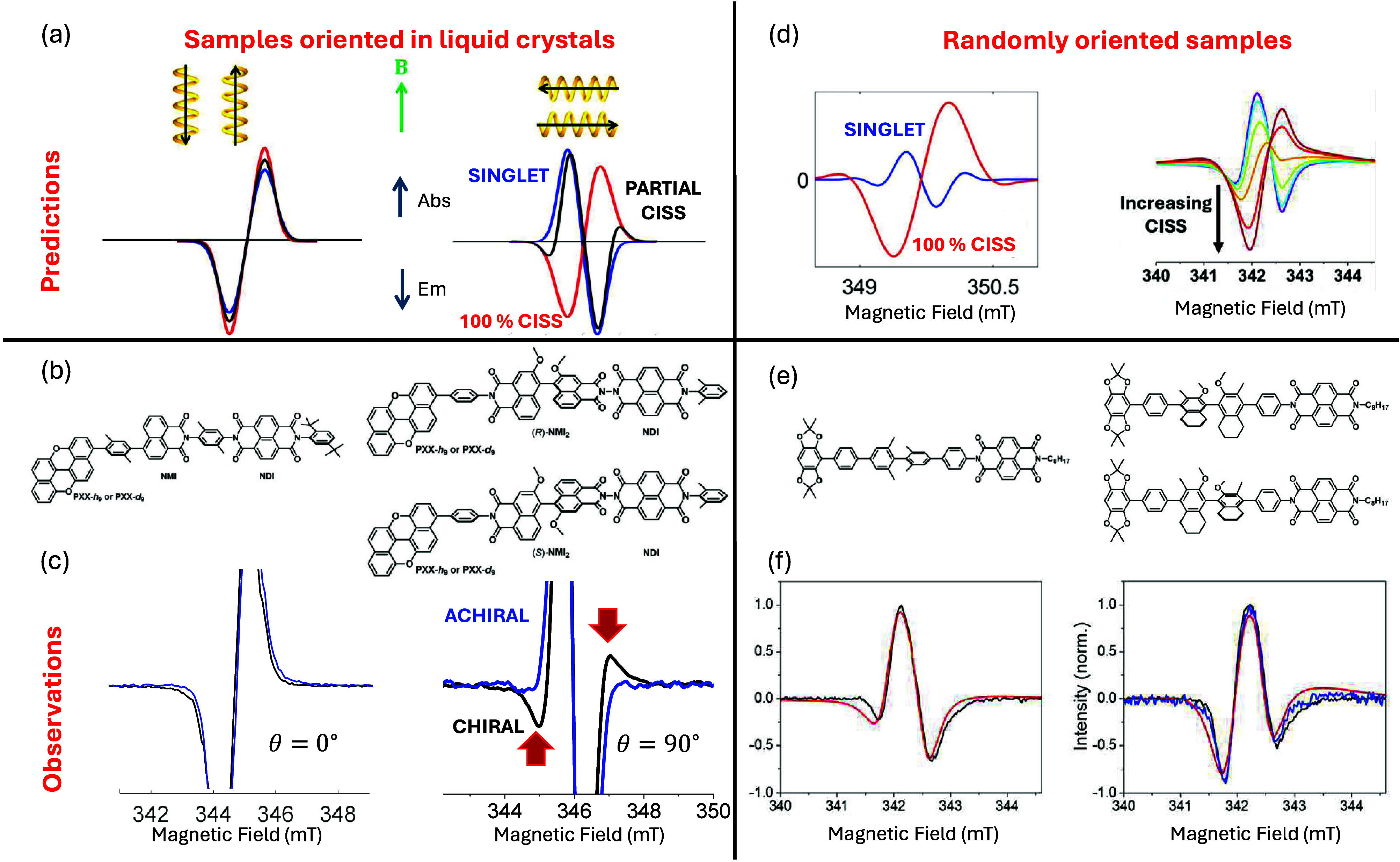
Unraveling CISS effect by time-resolved EPR. (a,d) Predicted spectra for samples oriented with liquid crystals (a)^18^ with the charge separation axis either parallel (left) or perpendicular (right) to the external field [only two peaks shown due to similar *g* tensors and line broadening] or in an isotropic solution (d) with different parameters as in refs 19, 44, and 45. (b) Molecular structures of reference achiral PXX-NMI-NDI system (left) and of chiral PXX-NMI_2_-NDI. Here PXX = peri-xanthenoxanthene, NMI = naphthalene-1,8-dicarboximides, and NDI = naphthalene-1,8:4,5-bis­(dicarboximide). (c) Corresponding experimental observations on protonated chiral PXX-NMI_2_-NDI (averaged on the two enantiomers) and reference achiral PXX-NMI-NDI system.^18^ (e) Molecular structures of the reference molecule (left) and of DB_χ_A enantiomers (right) where *D* = 2,2,6,6-tetramethyl-[1,3]-dioxolo­[4,5-*f* ]­[1,3]­benzodioxole, *B*_χ_ = (*R*)- or (*S*)-2,2′-dimethoxy-4,4′-diphenyl-5,5′,6,6′,7,7′,8,8′-octahydro-1,1′-binaphthalene, and *A* = naphthalene-(1,4:5,8)-bis­(dicarboximide) studied in a randomly oriented solution. (f) Corresponding X-band spectra (black, blue lines)^19^ and fit (red) with a CISS contribution of 38%. In the right panel, black and blue lines are the (*S*) and (*R*) enantiomer. Reproduced (adapted) with permission from ref 19. Copyright 2024 American Chemical Society.

In contrast, the perpendicular orientation (θ = 90°) is the most informative because it leads to an initial excess population on |*T*_+1_⟩ and |*T*_–1_⟩ which is opposite to the singlet case. As a result, by comparing singlet and 100% CISS states, we expect the two most intense peaks in the TREPR spectrum to be reversed (blue vs red lines in the right part of Figure 3a). Conversely, the singlet case is isotropic and shows the same initial population on |Φ_*A*_⟩ and |Φ_*B*_⟩ independently of θ.

A partial CISS polarization will in general lead to intermediate situations [|χ| < π/2 in eq 3], in which the intensity of the EPR signal results from the sum of two contributions from the singlet and 100% CISS states, respectively. For θ = 90°, this implies partial cancellation of the peaks, which occur at the same resonance fields. However, the blue and red traces in the right part of Figure 3a are characterized by a significantly different line shape.^18^ As a result, the CISS line is broader and, when combined with the singlet contribution, yields lateral wings of opposite sign compared to the leading signal (black trace).^18^ Note that this predicted behavior is qualitatively general and it does not depend on the specific parameters in the Hamiltonian [these can only affect the precise value of the CISS efficiency], but only on the general form of the anisotropic (dipolar) spin–spin interaction in the oriented sample. Moreover, the contribution of the two enantiomers to the EPR intensity is equivalent to that of two molecules rotated by 180°, already included in the above considerations. Hence, the two enantiomers and a racemic mixture give exactly the same EPR spectrum. A final remark is in order: these experiments do not give access to the relative phase between |*S*⟩ and |*T*_0_⟩ components in eq 3, but only to the squared absolute component of |*T*_0_⟩,  sin^2^χ/2. In addition, they do not discriminate the superposition state of eq 3 from a mixture (with reduced coherences) between |*S*⟩ and |*T*_0_⟩ [slight differences in the spectrum could arise at different values of θ but they would be hardly distinguishable in an experiment].

To experimentally demonstrate this idea, a chiral PXX-NMI_2_-NDI molecule was synthesized, together with its achiral PXX-NMI-NDI reference system^18^ [Figure 3b]. The NMI_2_ bridge has axial chirality and is based on the well-known sterically hindered binaphthols. Photoexcitation of the PXX donor leads to a two-step fast ET to produce the charge separated PXX^+^-(NMI_2_)-NDI^–^ state. Samples of each enantiomer of PXX-NMI_2_-NDI and of PXX-NMI-NDI were each aligned using 5CB and spectra were recorded for different orientations of the external field compared to the charge separation axis, thus realizing the conditions illustrated in Figure 3a. Results for the two enantiopure samples (black and red) as well as for the achiral reference (blue) are shown in Figure 3c. While the spectra for the chiral and axhiral derivatives are identical in the parallel configuration, they display the predicted lateral wings in the perpendicular orientation, which therefore represent a clear demonstration of the CISS effect on isolated molecules, with the CISS contributing about 50%.

As mentioned in the previous section, it was predicted^44^ that CISS effect should be visible even on an randomly oriented solution of chiral molecules, in the presence of an anisotropic spin–spin interaction between the two spins [Figure 3d]. The possibility of unambiguously unveiling the effect is less trivial, especially in the presence of mixed singlet/tripled excited state precursors, as evidenced by attempts on a CdSe-oligopeptide-C_60_ molecule.^46^ Moreover, a systematic study^45^ showed that evidence of the effect can change depending on the system eigenstates (and hence on the *g* tensors and on the microwave frequency). Nonetheless, these predictions were recently confirmed with observation of CISS in hole transfer in a randomly oriented solution of DB_χ_A molecules^19^ [Figure 3e]. The compared X-band spectra of the achiral (left) and chiral (right) molecules are reported in Figure 3f, with the occurrence of CISS demonstrated by the increased intensity of the minimum at low field compared to the other one.

## Current Status of the Theory

Although many ideas were put forward to model photoemission, transport, and other experiments involving surfaces,^1,8,36^ much less attention has been so far devoted to ET. This purely molecular situation is ideal for comparing with experiments and testing different theoretical models for CISS, including various factors that could play an important role, such as electron–electron interactions and both coherent and incoherent coupling with vibrational modes.

The TREPR experiments presented above highlight the presence of a triplet component in the initial state of SCRP after PET through a chiral bridge. However, they leave at least two important questions still open:

(i) Does this state display a local spin polarization on D/A or only a spin coherence?
(ii) Is the electron spin filtered or polarized by the chiral bridge, i.e., does the ET rate depend on the spin state of the transferred electron?

Indeed, both of these points are determinant for possible quantum applications.^6^ For instance, the occurrence of local spin polarization would enable high-temperature initialization, while spin filtering would convert spin polarization into charge separation with a substantial impact on the spin readout. Below, we discuss models developed so far for the CISS effect in PET and how they address these questions.

### Models without Inclusion of the Chiral Bridge Electrons

The first class of models does not include explicitly the electronic degrees of freedom of the bridge but only considers charge states. The simplest case is with two states corresponding to either two electrons on the donor or one electron on the donor and one electron on the acceptor. In standard (spin-independent) ET, these states interact via a *real* charge transfer potential independent of nuclear coordinates (the so-called Condon approximation). This interaction leads to a transfer of the electronic charge without affecting its spin state.

Fay proposed^42^ to include the effect of spin–orbit coupling (SOC) in the chiral bridge by adding an imaginary term to the charge transfer potential. This yields a *rotation* of the transferred spin *s*_2_ during ET. The resulting state of the SCRP (starting from a singlet on the donor after photoexcitation), is a superposition of |*S*⟩ and |*T*_0_⟩ of the form

6a $$|\psi \left(\chi ,n\right)\rangle =\cos\frac{\chi }{2}|S\rangle +i\;\sin\frac{\chi }{2}|T_{0}\left(n\right)\rangle$$

6b $$=e^{i\chi /2}|↑↓\left(n\right)\rangle -e^{-i\chi /2}|↓↑\left(n\right)\rangle$$

Here the axis **n** and the rotation angle χ are dictated by the SOC vector and by the ratio between imaginary and real components of the charge transfer potential.^42^

It is worth noting that this state does not show local spin polarization (*p*_*DA*_ = 0), since in the coupled basis [eq 6a] the coherence between |*S*⟩ and |*T*_0_⟩ is purely imaginary or, equivalently, |α|^2^ = |β|^2^ in the factorized basis of eq 6b. This appears in contrast with experimental observation of spin polarization in electron transfer on photosystem-I using a spintronic device.^47^

Starting from this model, Fay and Limmer considered a two-step ET process,^48^ where local polarization arises due to the combined effect of the SOC (in the form of a complex charge transfer potential) and of the spin–spin exchange interaction 2*J* **s**_1_ · **s**_2_ between the spin on the donor *s*_1_ and the one of the transferred electron *s*_2_ [Figure 4a]. In particular, SOC is assumed to act in the first ET step, as in the previous model (with rate *k*_ET1_), while the exchange interaction acts only in the intermediate state. Then, the second ET step occurs with a rate *k*_ET2_ and is spin-independent. In the coupled *S*–*T*_0_ basis, the exchange interaction between the donor and the transferred spins is diagonal and hence the related time-evolution introduces a time-dependent phase into the purely imaginary coherence of eq 6a, thus making *p*_DA_ nonzero. Fay and Limmer found an upper bound of 1/2 for *p*_DA_, for a certain relative values of *J*, *k*_ET2_, the ratio between imaginary and real part of the charge transfer potential and of a shift term emerging from master-equation theory.^48^ In practice, the highest CISS efficiency is obtained for intermediate values of *k*_ET2_/2*J* [see Figure 4b]. Indeed, too small values of *k*_ET2_/2*J* (top panel) lead to spin polarization averaging to 0 before the second ET step, while too large values do not allow the exchange to build up a sizable spin polarization before the second ET step (bottom panel).

**Figure 4:**
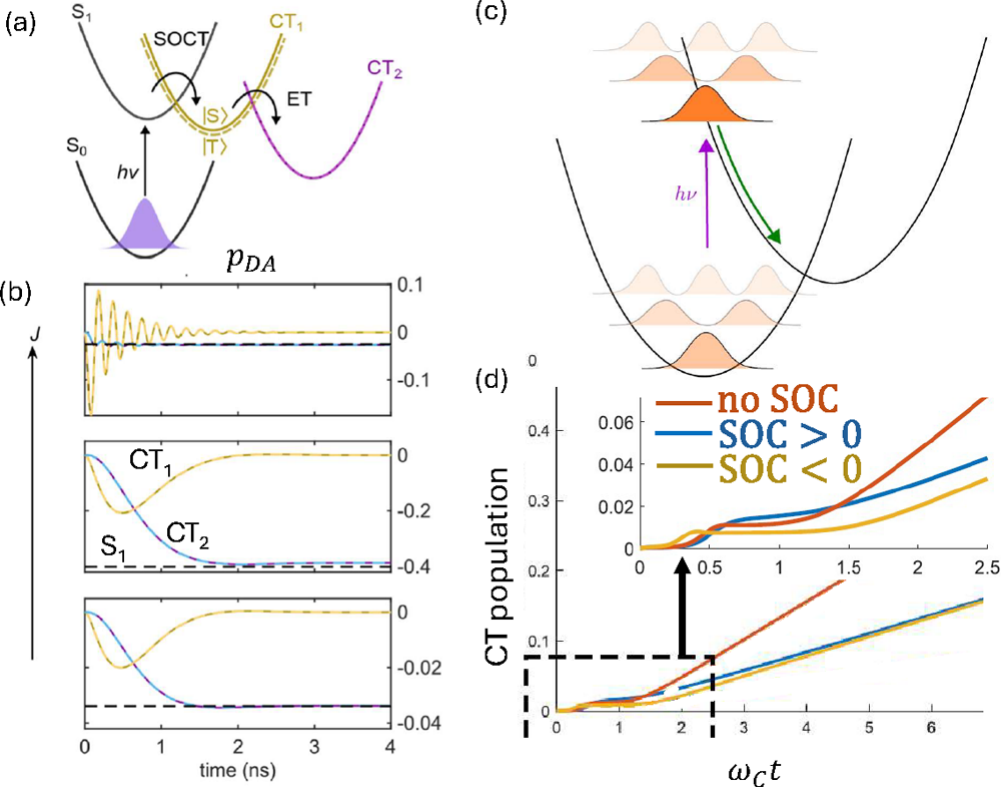
Modeling for CISS in ET with the chiral bridge as a complex charge-transfer potential. (a) Two-step process proposed by Fay and Limmer,^48^ with SOC acting only in the first step and spin–spin exchange interactions only in the CT_1_ intermediate charge state. The second step (from CT_1_ to CT_2_, with one electron on the donor and one on the acceptor) is spin independent. (b) Results for the spin polarization *p*_DA_ = (*p*_D_ – *p*_A_)/2 dynamics for each charge state for decreasing values of the exchange interaction 2*J* from top (*J*/*g*_*e*_μ_*B*_ = 100 mT) to bottom (*J*/*g*_*e*_μ_*B*_ = 1 mT). Reproduced (adapted) with permission from ref 48. Copyright 2021 American Chemical Society. (c,d) ET model from ref 49 beyond the Condon approximation. (c) A fast photoexcitation changes the electronic state but not the nuclear coordinates. As a result, the vibrational degrees of freedom are placed in an out of equilibrium state on the excited surface. (d) Population of the charge transfer state without spin–orbit coupling (orange line), and for two opposite signs of the SOC (blue and yellow lines, respectively), with the inclusion of decoherence. A spin filtering effect emerges at short times (see zoom in the inset) and then vanishes. Reproduced (adapted) with permission from ref 49. Copyright 2022 AIP.

From the Marcus theory for *k*_ET2_, the temperature dependence of the spin polarization is also derived. While the trend is in agreement with measurements on photosystem-I,^47^ the observed spin polarization exceeds the value of 1/2 predicted by the theory.

In summary, the models introduced in refs 42 and 48 yield a local spin polarization in two-step ET processes. The same treatment was recently applied to charge recombination, showing the possibility of spin polarization only in a two-step hopping process,^50^ in perfect analogy with the forward reaction.

We finally remark that this mechanism does not lead to a spin filtering, i.e., the charge is always completely transferred from D to A [see Figure 4a].

The possibility of a spin filtering in PET (i.e., of a spin dependent charge-transfer rate) was pointed out by Wu et al. in refs 49 and 51–53 . This filtering arises by considering the coupled electron–nuclear dynamics in the presence of a complex charge transfer potential which breaks the Condon approximation (i.e., depends on the nuclear coordinates). In this scenario and if mirror-symmetry is broken (as in chiral systems), nuclei are subject to a *Berry force* equivalent to an effective magnetic field on a charged particle, which is opposite for the two spin states of the electron.^54^ This, in turn, yields a different propagation of the two spin components and hence different electron-transfer rates. The Authors compute these spin-dependent rates using nonequilibrium Fermi Golden rule after having initialized the system in an out-of-equilibrium configuration of the nuclear degrees of freedom which mimics photoexcitation [see Figure 4c]. Then, a system prepared with a given spin polarization displays different dynamics depending on the sign of the spin–orbit coupling [blue vs yellow curves in Figure 4d], mostly at short times (inset). This corresponds to different ET rates for the two spin components, i.e., to spin filtering. The main features controlling this spin-filtering effect are (i) a complex charge transfer potential between electronic states, dependent on the nuclear coordinates, (ii) a coupling between electrons and vibrations, whose dynamics must be explicitly included in the model beyond the Born–Oppenheimer approximation, and (iii) an initial out-of-equilibrium state displaying coherence between vibrational states. The effect recalls a similar result obtained in the gas phase near a conical intersection,^51,52^ i.e., a region in the set of nuclear spin configurations in which two or more potential energy surfaces intersect. This enhances the effect of the coupling between electronic and nuclear degrees of freedom and yields a spin-dependent scattering of an electron wave packet. In condensed matter, the effect of friction must be taken into account by coupling vibrational modes to a bath. This yields decoherence of the nuclear spin degrees of freedom, thus making the computed spin selectivity only a transient effect. In particular, the charge dynamics is different for the two enantiomers only for a few 1/ω_c_ (ω_c_ being the frequency cutoff of the vibrational mode), as shown in Figure 4d.^49^ Nevertheless, the effects on spin selectivity of anharmonic potential and/or contributions beyond first order in the transition rates should still be investigated.^49^ In general, these results point to a crucial role played by the explicit inclusion of nuclear motion.

### Including the Chiral Bridge Electrons as Active Players

In the aforementioned models, the bridge was treated as an effective charge-transfer potential, providing the effect of the SOC through its nuclear degrees of freedom (and possibly their related motion). However, its internal electronic degrees of freedom were not taken into account. As a result, the chiral bridge does not interact explicitly with the transferred electron. To go beyond this description, some of us recently put forward a minimal many-body model with explicit inclusion of these internal degrees of freedom of the bridge.^55^ At equilibrium, the bridge is described by a Hubbard half-filled chain of *N* = 4 electrons and 4 orbitals with nearest-neighbors hopping integrals *t*, on-site Coulomb repulsion *U* and SOC as a spin-dependent next-to-nearest neighbor imaginary hopping of strength λ, as in ref 56 [see scheme in Figure 5a]. To investigate the role of electron–electron correlations, a regime of small *t*/*U* is considered, in which the equilibrium ground state of the bridge is a singlet, split by an exchange interaction *J* = 4*t* ^2^/*U* from the first excited multiplet. As depicted in Figure 5a, the first and last sites of the chain are linked to the donor (initially prepared with two electrons in a singlet state on two different orbitals, as just after photoexcitation) and to the acceptor (modeled by an initially empty orbital).

**Figure 5:**
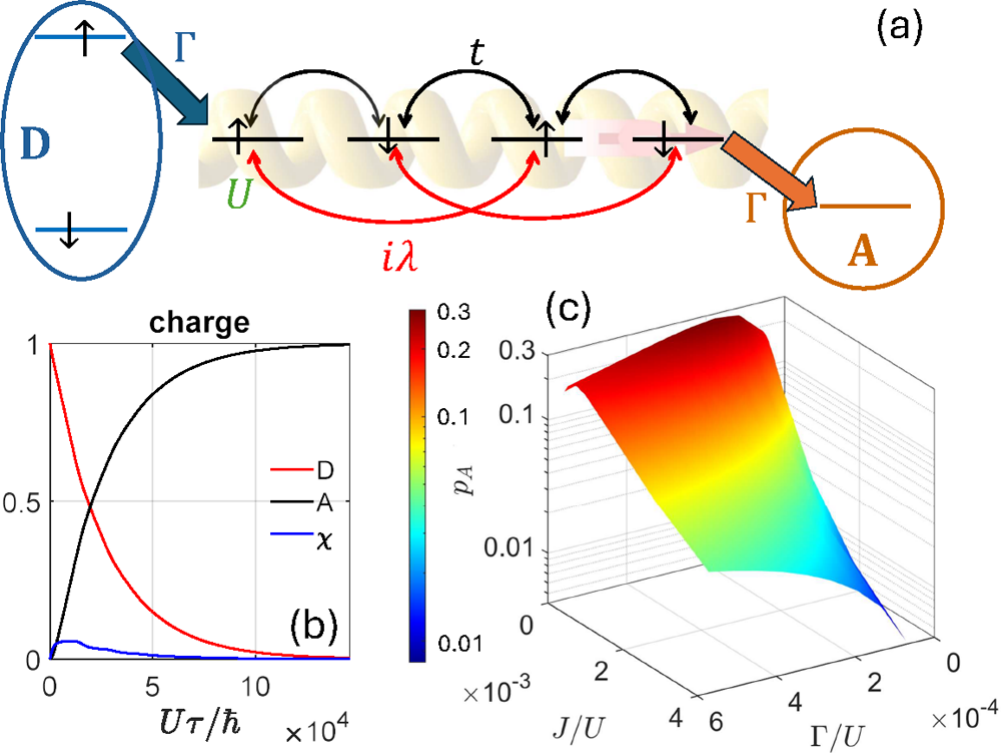
(a) Many-body model for CISS in Electron Transfer from ref 55. The chiral bridge is described by a Hubbard Hamiltonian with nearest neighbor hopping parametrized by *t*, next-to-nearest neighbors SOC of strength λ and on-site Coulomb repulsion *U*. Donor (D) and acceptor (A) are coupled via incoherent spin-conserving jump operators, included in the Redfield equation with rates Γ. (b) Population of the different orbitals during the simulated charge transfer dynamics: donor excited orbital (red), acceptor (black) and sum of the sites on the bridge (blue) referred to the equilibrium (*N* = 4) population. (c) Spin polarization *p*_*A*_ accumulated on the acceptor after ET, as a function of Γ and of the exchange coupling *J* = 4*t* ^2^/*U*, for λ = 6.25 × 10^–4^ *U*.

ET is described by incoherent spin-conserving jump operators from the donor excited orbital to the bridge and from the bridge to the acceptor. During the ET, the charge is transferred from the donor to the acceptor [see Figure 5b], after populating states with (*N* + 1)–electrons on the bridge. In parallel, a spin polarization *p*_A_ is accumulated on the acceptor, arising from the combined coherent Hamiltonian dynamics on the bridge and incoherent transfer (with rate Γ) from D and to A. In particular, to make SOC effective and hence establish a spin polarization, a comparable energy scale must be present in the molecular spectrum with *N* + 1 electrons, provided here by the *intrabridge* exchange interaction *J* [the electron on the donor does not give any effect in this model]. A sizable polarization then arises on A from the interplay between *J* = 4*t* ^2^/*U*, λ and Γ and is maximized when these parameters assume comparable values [Figure 5c]. This implies, in particular, a small *J* ∼ λ, while polarization vanishes in the large *t*/*U* limit. Remarkably, *p*_*A*_ reaches significant values (∼0.3) for a reasonable SOC of a few meV, typical of light atoms in organic molecules.

The model proposed in ref 55 does not predict different transfer rates for electrons with opposite spin polarization, analogously to ref 48. In contrast, this effect emerges in ref 49 at short times from including coupled electron–nuclear dynamics. The possibility of an active role of *polarons* (i.e., mixed Fermionic-bosonic excitations) in the spin polarization mechanism was already suggested by explicit inclusion of a local mode in the bridge Hamiltonian in ref 55, but the mechanism deserves further investigation.

### Analogies with Models for CISS in Thin Films and Devices

These latter results are consistent with previous reports of modeling transport experiments and can be naturally extended to this situation. In particular, the importance of electron–electron interactions in amplifying the effect of the SOC in transport through chiral junctions was pointed out in refs 56 and 57. The role of polarons and in general of electron–vibration interactions (possibly mediating electron–electron correlations) was also investigated,^58–61^ and the importance to go beyond the Born–Oppenheimer approximation was underlined.^1,51,52^ A significant spin polarization of the electronic current was also obtained as a result of nuclear dynamics and Berry force^62^ in a model for transport through a two-orbital molecular junction for large voltages.

In general, in both PET and transport, the small magnitude of the intrinsic SOC of organic molecules requires the theory to go beyond a single-electron description^35,56,58,63–65^ to enhance its effect and yield a sizable spin polarization. While accounting for these complex factors in the bulk becomes very demanding, the minimal *molecular* model of ref 55 could be extended to investigate the ET dynamics in the presence of polarons or, in general, low-energy vibrations in different coupling regimes, which were pointed out as crucial for establishing a spin polarization in transport.^58,63^

Several caveats should be considered when comparing models for CISS in PET with transport or photoemission. Although these different processes likely manifest the same underlying phenomenon, their distinct behaviors must be considered in theoretical modeling. Transport measurements are performed by embedding the chiral molecules between two electrodes. One of the two contacts is ferromagnetic, and the other is nonmagnetic. Then, *I*–*V* curves are detected for both bias polarities.^1,66^ In one case, the ferromagnetic layer acts as a polarizer, thus injecting a spin-polarized current through the chiral junction. Then, a different steady-state current is measured, depending on the initial spin polarization, thus indicating a spin-filtering effect. By reversing the bias, the ferromagnetic layer acts as an analyzer for the spin polarization induced by the chiral molecules in the initially unpolarized current. This second configuration is similar to PET and photoemission, where electrons are excited with no preferential spin orientation, and spin polarization is detected in the outcome. This gives direct access to the spin polarization but does not imply a filtering effect.

An important difference between transport and PET/photoemission lies in the nature of the studied phenomena: steady-state current versus transient processes. In steady-state transport measurements, charge could accumulate on the bridge during an initial transient step, potentially leading to charge polarization contributing to the subsequent filtering effect.^67^

Additionally, in PET experiments, electron transfer strongly depends on several molecular parameters that require precise optimization. In particular, long chiral molecules, such as polymers or DNA up to 10–100 nm, cannot be studied because the PET becomes very inefficient.^68^ Furthermore, in ET, the electron continues to interact with the positive charge left behind and with its electronic spin, an essential ingredient in models of refs 48 and 50. In contrast, in transport measurements, the electron reaches the counter electrode and no longer interacts with the donor molecule. All in all, combining these similarities and differences could provide further insight into the phenomenon.

## Steps Forward

Theoretical efforts should be combined with targeted experiments to shed light on open issues on the polarization mechanism and on the charge-separated spin-pair state. To date, comparisons have been made only between a chiral molecule and its achiral counterpart in liquid crystals, where full orientation is not possible.^18,19^ In particular, TREPR experiments conducted to date on D/A dyads demonstrated the presence of a triplet component in the SCRP after PET through a chiral bridge. However, they do not yet provide evidence for local spin polarization, meaning they do not distinguish between the models proposed in ref 42 or in refs 41, 48, and 50, which involve either an imaginary or a real coherence between |*S*⟩ and |*T*_0_⟩. Another important open question concerns the potential for achieving spin filtering in the ET process, which has yet to be demonstrated.

The use of fully oriented, i.e., poled, samples would provide much more information on the CISS effect in PET. In this case, the most informative TREPR experiments would be with significant Δ*g* between the two ions and with the chiral axis parallel (or antiparallel) to the magnetic field, giving rise to very different response for each enantiomer (see Figure 2), i.e., extracting a sort of asymmetric factor.

Full orientation can be achieved using single crystals, but this has advantages and drawbacks. On the one hand, EPR measurements on crystals are well established and provide high precision in the determination of magnetic parameters.^69–72^ On the other, solubility issues, often solved by adding bulky aliphatic groups, reduce the size and quality of crystals. Moreover, photoactive molecules must be well separated in the crystal lattice to prevent the occurrence of an intermolecular ET.

An alternative approach makes use of thin films, where all molecules are isooriented with respect to the substrate. Molecular orientation and dispersion in a suitable host can be controlled in a layer-by-layer growth, e.g., through Langmuir–Blodgett deposition.^73^ However, a key requirement for this method is that the film must be thick enough to provide sufficient absorbance and enable the detection of EPR signals. The necessary thickness depends on factors such as the extinction coefficient of the molecule, the efficiency of electron transfer, and spectroscopic parameters like the EPR line width and the anisotropy of the *g*– and *A*–tensors. Films likely need to exceed tens of monolayers in thickness to overcome the EPR sensitivity challenges.

One way to mitigate this problem is to utilize optically detected magnetic resonance (ODMR) to probe the relevant spin states.^74,75^ Since this technique can read out the spin information using fluorescence emission, the sensitivity of the measurements can approach the single molecule level.

Another important point that deserves both theoretical and experimental investigation is the effect of the temperature. Indeed, the temperature dependence of the CISS effect could pinpoint the role of vibrations in triggering not only the charge transfer, but also the polarization dynamics, as pointed out in many theoretical models (see, among others, refs 49, 64, and 76). In particular, low energy modes have been suggested as crucial to observe a sizable polarization and are also the most influenced by temperature variations.

Atomistic modeling of the chiral bridge to investigate its contribution to CISS requires validation through spectroscopic quantification of the CISS efficiency in different types of chiral linkers, varying in chirality type (stereogenic center, axial, planar, or helical) and electron transport mechanisms. Simultaneously, the optimization of the PET process and the lifetime of the charge-separated state, which is necessary to detect CISS by TREPR, remains challenging.

Important information about the mechanism behind CISS could also be obtained by investigating the spin selectivity in transport through the same bridge. To achieve this, a well-controlled geometry of the molecule–electrode interface is essential. This can be accomplished by using dense self-assembled monolayers of long helical molecules, such as oligopeptides, functionalized with a tethering group. Unfortunately, the chiral linkers that have been widely employed and demonstrated high spin selectivity under an electric bias are not efficient for promoting PET even with the appropriate energy alignment of the donor/acceptor states.^68^

To elucidate the nature of the charge-transfer state and improve our theoretical understanding of CISS targeted experiments can be designed. For instance, additional information on the CISS state could be gained by starting from a triplet excited state precursor.^46^ In particular, evidence of a possible spin-filtering could emerge by preparing the state with a population imbalance between |*T*_–1_⟩ and |*T*_+1_⟩ before ET (e.g., by blocking ET with an applied electric field). Indeed, an external electric field provides an additional interesting tool to control both forward and backward reaction rates. This could be used to study the CISS effect also in the recombination process, predicted for instance in ref 50. An open question is what can we say about recombination of the CT state to the low-lying triplets? If this process is too slow, then electric fields or light pulses could be used to favor recombination.

Last but not least, one could design interesting experiments with a third spin (either an electronic spin qubit or a nuclear spin) acting as an *observer* probe of the state of the acceptor/donor spin (see subsection below).

### Three-Spin Experiments

A third electronic or nuclear spin attached to a dyad could probe the state of the acceptor/donor spin. The advantage of this approach is that the probe is not directly involved in the phenomenon (CISS in ET) that we want to detect and exists even before charge separation. To this aim, we could exploit either an electronic or nuclear spin at the donor/acceptor sites. For instance, TREPR experiments on a qubit in a fully aligned sample would give clear signatures of the nature of the charge-separated state.^44^ The presence of an interaction between the qubit sensor and the donor/acceptor indeed yields splittings in the absorption peaks reflecting the polarization state of the probed site.^44^ In addition, proper spin polarization transfer experiments from the acceptor to the linked third spin can be designed.^44^

Another promising probe of the CISS is provided by nuclear spins like protons. Indeed, ^1^H nuclei are naturally present in SCRPs and coupled by hyperfine interactions to D/A, with no need of further synthetic efforts. Moreover, the eigenstates of the radical pair are only weakly perturbed by these probes, thanks to the very large difference in Zeeman energies between electronic and nuclear spins. Indeed, the mixing between nuclear and electronic spins interacting through an hyperfine interaction $AS_{A}\cdot I$ is of the order of $A/g\mu_{B}B\ll 1$ . Conversely, a linked electronic spin would mix via a term of the order of *D*/Δ*g*μ_*B*_ *B*. Even with D≈A , the difference of about 3 orders of magnitude in the denominators makes an electronic probe much more entangled with the SCRP.

These nuclei can be probed by time-resolved electron–nuclear double resonance (TR-ENDOR), as shown for the D/A system P–700^+^A_1_^–^ in the photosystem I protein complex.^77^ The ENDOR spectra of the SCRP are sensitive not only to the hyperfine interaction between the nuclei and the unpaired electrons but also to the coupling between the electrons. The resulting ENDOR spectra show, like the TREPR spectra, positive and negative intensities. The sensitivity advantage of ENDOR indirectly detecting the NMR spectra of paramagnetic species via EPR comes here at the expense of highly complex spectra, which can make their interpretation in terms of local electron polarization, i.e., distinction between absence or presence of CISS, difficult.

A more direct way to assess the nuclear spins as probes for the electronic state of the SCRP is to perform broadband NMR experiments, exploiting Hahn echo pulse sequences to obtain the spectrum as a function of frequency. Indeed, with an optimized experimental setup, samples suitable for EPR investigations can also be studied with NMR. Since the electron spin magnetic moments induce large shifts (≈ MHz) on the nuclei, it is possible to exploit lower resolution in frequency and shorter RF pulses with respect to standard NMR setups, while covering a large frequency range (broadband). A drawback of NMR compared to EPR is, however, the lower sensitivity, which requires the use of rather large samples. In addition, nuclear spins require longer manipulation times, even if experiments can still be done within the lifetime of the RP by state-of-the-art ENDOR/broadband NMR. In ref 44, it was shown that the NMR absorption signal of a nuclear probe interacting with the donor/acceptor reflects the nature of the charge-separated state, being sensitive to the state of the acceptor. These NMR experiments were originally proposed for fully oriented dyads. Here we propose performing NMR experiments for CISS detection also on an isotropic solution. Figure 6 shows the NMR spectra as a function of frequency, probing nuclear transitions of a nuclear spin of 1/2, e.g., ^1^H, split by a hyperfine interaction with the acceptor.

**Figure 6:**
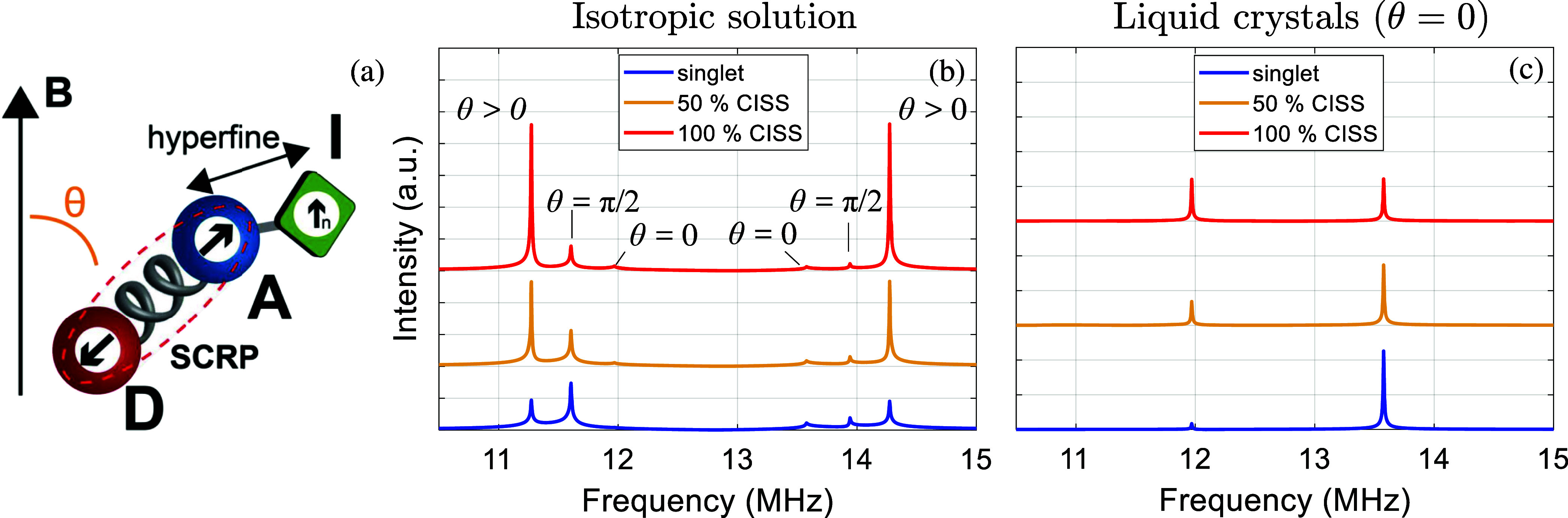
(a) Sketch of a three-spin system: the acceptor in the chiral D-χ-A SCRP also interacts with a nuclear spin I through isotropic hyperfine interaction. (b,c) NMR spectra as a function of frequency, probing nuclear transitions of a nuclear spin 1/2 (a ^1^H, with Larmor frequency ν_L_ = 42.58 MHz at 1 T) coupled by isotropic hyperfine interaction to the acceptor and prepared in a $\left(|↑\rangle +|↓\rangle \right)/\sqrt{2}$ state. Simulations consider samples containing both enantiomers of a D−χ–A dyad in an isotropic solution (b) or oriented with liquid crystals with the static field applied along the chiral/dipolar axis (θ = 0, c). Spectra show distinctive features characterizing the polarized charge-separated state with respect to a singlet state (blue lines), both in the presence of 100% (red lines) or 50% spin polarization (orange lines). Characteristic peaks for θ = 0 and θ = π/2 are labeled in (b). Parameters: *D* = 6.5 MHz corresponding to *r*_DA_ = 2 nm, A = 3 MHz, *g*_D_ = 2.004, *g*_A_ = *g*_D_ – Δ*g* with Δ*g* = 0.001, *B*_0_ = 300 mT, T_2_^*n*^ = 50 μs.

With a parameter set typical of dyads showing CISS (see the caption of Figure 6), a D−χ–A–I three-spin system is characterized by eigenstates where nuclear spin states are factorized from that of the two electronic spins (the same as in Figure 1). The hyperfine interaction between the ^1^H and the acceptor yet make the |Φ_*A*_⟩ and |Φ_*B*_⟩ superposition states slightly different depending on the state of the nuclear spin, i.e., $|\Phi_{A},{↑}_{n}\rangle =\cos\phi |S\rangle +\sin\phi |T_{0}\rangle \neq \;|\Phi_{A}^{\prime },{↓}_{n}\rangle =\cos\phi^{\prime }|S\rangle +\sin\phi^{\prime }|T_{0}\rangle$ .

Our simulations show that the coherent dynamics of the nuclear probe, initially prepared in the coherent superposition $\left(|{↑}_{n}\rangle +|{↓}_{n}\rangle \right)/\sqrt{2}$ by a π/2 pulse, strongly depends on the nature of the photoinduced charge-separated state, even in an isotropic solution (Figure 6b). To illustrate this, we start by considering a sample aligned with LCs with the static field *B*_0_ applied along the chiral/dipolar axis (θ = 0), as in Figure 6c. We focus on purely nuclear transitions (i.e., between eigenstates with substantially the same electronic spins state), whose energy, however, depends on the state of the SCRP due to the hyperfine interaction. If the dyad is in a generic initial state with components on both eigenstates |Φ_*A*_⟩ and |Φ_*B*_⟩ (see Figure 1), the NMR spectrum shows two peaks, corresponding to the nuclear transitions |Φ_*A*_, ↑_*n*_⟩ ↔ |Φ_*A*_^′^, ↓_*n*_⟩, and |Φ_*B*_, ↑_*n*_⟩ ↔ |Φ_*B*_^′^, ↓_*n*_⟩. Let us now examine the situation in the presence or absence of CISS. If the SCRP has undergone CISS with 100% efficiency, we observe two peaks of similar intensities (red lines). Instead, an initial singlet state of the SCRP is close to being an eigenstate of the three-spin Hamiltonian and hence only the |Φ_*A*_, *↑*_*n*_⟩ and |Φ_*A*_^′^, ↓_*n*_⟩ states have a non-negligible population. Thus, for not too large Δ*g*, the NMR spectrum of the nuclear probe provides a clear fingerprint of CISS even in the presence of a longitudinal field (Figure 6c). The larger Δ*g*, the more the red and blue peak at 12 MHz in Figure 6c become similar and the CISS effect harder to be observed. This is analogous to what is expected from TREPR in the same experimental configuration: for small Δ*g* but sufficiently separated peaks one would expect an intensity difference between singlet and CISS precursors, which however vanishes if the peaks get too broad or if Δ*g* is increased (as in Figure 2). It is worth stressing that in NMR the Δ*g* only governs the relative intensity of the peaks resulting from different SCRP states, while their separation in frequency only depends on the hyperfine coupling A and on the direction of *B*_0_ with respect to the chiral/dipolar axis.

As soon as the static field *B*_0_ is no longer applied along the chiral/dipolar axis, two additional peaks emerge (labeled as θ > 0 peaks in Figure 6b), reaching their maximum intensity at θ = π/2. They correspond to the nuclear transitions |*T*_+1_, *↑*_*n*_⟩ ↔ |*T*_+1_, *↓*_*n*_⟩, and |*T*_–1_, *↑*_*n*_⟩ ↔ |*T*_–1_, *↓*_*n*_⟩ and are an unambiguous signature of a polarized state of the dyad. These eigenstates have in fact practically zero populations and coherences for a singlet charge-separated state, and thus, these transitions cannot be observed in this case. In addition, only the transition probabilities are affected by the direction of the applied field *B*_0_, while the energy gaps between the involved pure triplet states remain unaltered. This ensures that in an isotropic solution the differences in the NMR spectral features of the nuclear probe between a singlet and a polarized state of dyad are further amplified, as, for instance, in the relative intensity between the θ = π/2 and the other peaks appearing when θ > 0 (Figure 6b). We have also verified that these same conclusions can be drawn even with a 50% CISS contribution (orange lines in Figure 6), since the polarized and singlet states still yield NMR transitions with different intensities.

Another interesting three-spin experiment to investigate CISS is based on transferring polarization from donor/acceptor to an additional observer spin, such as that of a permanent radical R^•^. A possible scheme based on coherent pulses was proposed in ref 44. Alternatively, polarization transfer could be realized by exploiting natural interactions in the three-spin Hamiltonian of a D^+•^BA^–•^R^•^ after photoexcitation. This mechanism was already reported in ref 78 for a compound with an achiral bridge consisting of a 4-aminonaphthalene-1,8-dicarboximide. After rapid charge separation, the singlet radical pair is converted to a triplet by intersystem crossing. Charge-recombination is also spin-selective, and the different rates for singlet and triplet recombination yield an opposite spin polarization of the radical. Exploring the same effect in the presence of a chiral bridge would provide further insight into the phenomenon.

In principle, CISS can also be probed by transferring spin polarization from the electron spins to nuclear spins having much longer relaxation times. Dynamic nuclear polarization (DNP) techniques are widely employed to spin polarize nuclear spins to enhance their detection sensitivity. Often high-power microwave sources are used to polarize the electron spins prior to polarization transfer; however, a variety of pulse techniques are also being employed with a good effect. For example, nuclear spin orientation via electron spin locking (NOVEL) was first proposed in 1988,^79^ and is now being applied widely, see, e.g., ref 80. In the NOVEL technique, polarization is efficiently transferred from electrons to nuclei using a rotating frame/lab frame Hartmann–Hahn matching condition ω_1*S*_ = ω_0*I*_, where ω_1*S*_ = γ_*e*_ *B*_1_ is the electron Rabi frequency and ω_0*I*_ = γ_*n*_ *B*_0_ is the nuclear Larmor frequency. Being able to transfer polarization created by CISS to the nuclei of the DB_χ_A molecule would not only provide another tool to investigate the CISS mechanism but also give a useful means of preserving the polarization for long times, thereby acting as a quantum memory.

### Quantum Applications

The CISS effect at the molecular level can be leveraged for a wide range of applications in quantum technologies. Clearly, developing schemes for these applications requires an improvement in our understanding of the phenomenon. In particular, we need to clarify if spins are polarized and/or filtered in the CISS effect at the molecular level and then tailor schemes for specific quantum applications based on that understanding. Below, we illustrate a few schemes highlighting their specific requirements in terms of spin polarization or filtering.

In general, the most promising approach relies on an architecture similar to that of the three-spin system discussed above. Molecular spin qubits^7^ can be linked to DB_χ_A molecules and then the CISS effect could be exploited for initialization, readout, and manipulations of the molecular spins.

In particular, *polarization* of the spin at the acceptor can be exploited to initialize the state of a linked qubit by a simple sequence of coherent microwave pulses,^6,37,44^ even at room temperature. This initialization procedure also opens the way for using the qubit as a local sensor of small magnetic fields at ambient temperature and with very high spatial resolution, where applications to medicine and biological processes are of great interest.^81^ Indeed, the Zeeman energy gap for a spin 1/2 in a typical Q-band applied field is small compared to *k*_B_ *T*, thus leading to a tiny thermal polarization (e.g., *P* ≈ 0.003 at 300 K) and to a resulting small signal-to-noise ratio in the measurement. Increasing the spin polarization (compared to thermal equilibrium) yields a proportional increase in the measured signal-to-noise ratio and hence in the sensitivity. Let us consider, for instance, a spin 1/2 subject to an external longitudinal field *B* which splits its eigenstates |*↓*⟩ and |*↑*⟩ by an amount Δ*E* = *g*μ_*B*_ *B*. According to the Ramsey scheme,^81^ the system is (i) prepared in |*↓*⟩, (ii) rotated into a superposition $\left(|↓\rangle +|↑\rangle \right)/\sqrt{2}$ by a resonant π/2 pulse, (iii) permitted to evolve for a proper interrogation time *t*, thus accumulating a phase difference Δ*Et*/*ℏ*, and finally (iv) detected after a second π/2 pulse. This leads to a signal ⟨*S*_*z*_⟩ ∝ *P* cos Δ*Et*/*ℏ*, where *P* is the initial polarization of the sensor. Hence, increasing *P* by CISS increases proportionally the contrast in the detected oscillations, thus enabling one to probe small magnetic fields even at temperatures characterized by very small thermal polarization.

Even more importantly, CISS can give rise to a spin to charge conversion mechanism which potentially enables read out of the state of an individual spin by borrowing techniques used in the semiconductor technology.^6^ Indeed, while a single spin interacts very weakly with external fields and hence is hardly detectable, the displacement of an electron charge in a DA pair (if allowed only for one spin orientation) can be probed much more easily. For instance, the two charge configurations could act as a gate voltage on a quantum dot in a single-electron transistor. As a result, a current flowing through the dot can be associated with only one of the two charge states. The important step is how to link such a charge state to the state of the spin qubit that we want to readout. A first possibility, proposed in ref 6, relies on spin *filtering*. Let us consider an example consisting of a DB_χ_AQ molecule with the qubit (Q) linked to the acceptor. At the readout stage (when the state of the radical pair is charge separated, with an unpaired spin on A), the state of Q is swapped to A. Then, charge recombination is induced by an external stimulus (such as an electric field or light), but due to spin filtering, this only occurs for one of the two spin projections along the chiral axis (due to CISS). Hence, the charge state of DA is associated with the former state of the qubit, allowing for projective readout.

Other schemes for the readout relying only on spin *polarization* in PET (without filtering) could be conceived.

On the synthetic side, the applications discussed above require integration of molecular qubits into chiral dyads or triads. In this context, two main families of molecular qubits are promising: organic radicals and transition metal-based systems. Both offer structural reproducibility, atomic-scale spatial control, and modularity.^82,83^ Moreover, both can be deposited on surfaces via thermal evaporation and organized into supramolecular structures, a key requirement for solid-state device implementation.^84–86^ Organic radicals are relatively simple spin systems, making them easier to study from a magnetic perspective.^87^ They exhibit long coherence times^88^ and have been extensively investigated for photoexcited molecular applications,^89^ serving as excellent model systems. Conversely, transition metal complexes offer greater tunability of spin Hamiltonian parameters at the metal centers, including the spin manifold, *g*-tensor anisotropy, and hyperfine coupling.^90^ They exhibit excellent coherence times, with coherent manipulation demonstrated even at room temperature.^91^ Metallorganic synthesis enables the fine-tuning of the spin properties in metal complexes, facilitating the creation of multilevel qudits^7^ the attachment of organic chromophores for light-induced studies,^92^ and the coupling of multiple qubits to construct quantum gates.^71,93^ Notably, besides providing a platform for quantum information processing, linking a metal ion would open new possibilities to investigate CISS effect.^94^

## Discussion and Conclusions

We have highlighted recent developments and future directions for investigating the CISS effect at the molecular level. Direct access to the spin selectivity in photoinduced electron transfer can be gained by time-resolved EPR spectroscopy. Recent experiments have marked fundamental milestones in studying the effect by showing it for the first time in molecules dispersed in solution not bound to any surface. This paves the way to a deeper understanding of the phenomenon in a simplified situation in which chiral organic linkers are the only players in the game. Such a simplification has allowed for the development of the first microscopic models highlighting some crucial ingredients for the emergence of spin polarization in molecules with small spin–orbit coupling. In particular, these include spin–spin interaction between the electron sitting on the donor and the transferred one, electron–electron correlations, and coupling with vibrational modes, possibly beyond the Born–Oppenheimer approximation. Nonetheless, several questions are still open.

The molecular approach combined with molecular engineering offers the potential to understand and finely control the key molecular factors that govern CISS. Despite the significant progress made so far, there is still a long road ahead to establishing a comprehensive framework that can address the open questions in existing theoretical models, such as the distinction between spin filtering and spin polarization. In this respect, a major step forward would be absolutely orienting donor–acceptor molecules relative to the magnetic field direction, which still represents an experimental challenge. The implementation of three-spin experiments with an additional electron or nuclear spin as a probe of donor–acceptor polarization could also provide fundamental insights.

Ultimately, bridging molecular-scale investigations with experiments on chiral films deposited on surfaces is potentially important for fully understanding the subtle interplay between light, spin, and chirality and exploiting it for technological applications. The full comprehension of CISS at the molecular level will have broad implications across fields beyond quantum information, where electron spin control plays a key role, including spin- and optoelectronics, enantioseparation, chemical reactivity, and biology.
